# Mindfulness-based social cognition training (SocialMIND) versus psychoeducational multicomponent intervention for people with a first episode of psychosis: a study protocol for a randomised controlled trial

**DOI:** 10.1186/s12888-019-2206-4

**Published:** 2019-07-29

**Authors:** Roberto Mediavilla, Ainoa Muñoz-Sanjose, Beatriz Rodriguez-Vega, Carmen Bayon, Angela Palao, Guillermo Lahera, Pilar Sanchez-Castro, Eva Roman, Susana Cebolla, Alvaro de Diego, Juan Manuel Pastor, Maria Fe Bravo-Ortiz

**Affiliations:** 1 Psychiatry and Mental Health Group, Hospital La Paz Institute for Health Research (IdiPAZ), Madrid, Spain; 2 National University of Distance Education (UNED), Madrid, Spain; 3 La Paz University Hospital, Madrid, Spain; 4 Autonomous University of Madrid (UAM), Madrid, Spain; 5 University of Alcala (UAH), Alcala de Henares, Spain; 6 Mental Health Networking Biomedical Research Centre (CIBERSAM), Madrid, Spain

**Keywords:** Mindfulness, Social cognition, Social functioning, Real-life outcomes, Psychosis, Schizophrenia spectrum disorders, Psychological intervention

## Abstract

**Background:**

People who suffer a first episode of psychosis experience higher levels of distress and suffering. Early intervention programs combine pharmacological and psychosocial strategies that include different components, such as cognitive-behavioural therapy, psychosocial interventions, medication adherence, family psychoeducation, counselling, etc. Among the complementary approaches, mindfulness-based interventions help participants to cultivate a radical acceptance of their psychotic experiences within a person-centered framework. They show promising results for people with longer duration of psychosis, but there is still no evidence for people who have recently experienced their first episode of psychosis.

**Methods:**

The present parallel-group, single-blind (evaluator), randomised (1:1 ratio), controlled (versus active comparator), superiority, clinical trial will compare the effectiveness of SocialMIND on social functioning as measured by the Personal and Social Performance (PSP) scale. The active comparator will be a psychoeducational multicomponent intervention (PMI) that incorporates elements of early intervention programs that are effective for people who have suffered a first episode of psychosis. Both SocialMIND and PMI encompass eight weekly sessions, four bi-weekly sessions, and five monthly sessions. Changes in primary and secondary outcomes will be measured after weekly (8th week), bi-weekly (16th week) and monthly sessions (56th week), and 3 months after completing the intervention (68th week). Secondary outcomes include symptoms of psychosis, anxiety and depression, as well as indicators of general functioning. Tertiary outcomes are measures of social cognition, neurocognition, mindfulness, and indicators of inflammation and oxidative stress. A final sample of 80 participants is proposed to detect clinically significant differences in social functioning.

**Discussion:**

This is the first mindfulness-based social cognition training for people with psychosis. SocialMIND aims to generate changes in the real-life functioning of people who have experienced a first episode of psychosis, and to be at least as effective as a psychoeducational multicomponent program. Adherence to the interventions is a common problem among young people with psychosis, so several difficulties are anticipated, and some methodological issues are discussed.

**Trial registration:**

The trial was registered in ClinicalTrials.gov in October 2018 (NCT03309475).

## Background

People who suffer a first episode of psychosis (FEP) are frequently adolescents or young adults [1, 2] and the recovery rate is around 40% [3]. Optimising the treatment of FEP is a priority for mental health experts [4], and early intervention programs must combine both pharmacological and psychosocial strategies [5, 6]. These psychosocial components are medication review, treatment adherence, vocational or educational counselling, psychoeducation, social skills training, or cognitive behavioural therapy, among others. There are some ongoing studies in Denmark [7], Italy [8], the United Kingdom [9], and other countries in Europe and abroad [10, 11]. The results are promising in terms of treatment discontinuation, hospitalisations, symptoms reduction, global functioning and quality of life, but there are still some concerns about their cost-effectiveness [12, 13].

Other, more focal, interventions such as cognitive behavioural therapy or family psychoeducation have also shown good results for FEP [14]. However, empirical evidence is still limited, and additional studies are welcomed [15]. The diagnosis-evidence-based-practice symptom-reduction model might be far from the needs and realities of patients, as it considers the FEP as a mere indicator of a vulnerability to develop a given diagnosis; on the other hand, a framework that promotes an accommodation to living with mental vulnerabilities through building resilience in the social and existential domains might be more useful in the clinical practice [16]. Mindfulness-based interventions (MBIs) have increased exponentially over the past three decades and they are gathering empirical evidence for emotional distress in the general population [17], for medical conditions [18], and for psychiatric disorders such as anxiety, depression, addictive behaviour, and psychosis [19]. Mindfulness is defined as paying attention to the present moment, on purpose, and non-judgmentally [20]. To cultivate this ability, mindfulness trainees learn a series of informal practices and formal meditation techniques, and they are invited to share their experiences during the group sessions. Both a radical acceptance of the experience and the ability to disengage from it are two core mindfulness dimensions that develop with its practice, and they have been proposed as new ways of coping with suffering for people with psychosis [21]. There is limited evidence regarding the effectiveness of MBIs for people with FEP, and no randomised controlled trials have yet published their results. Recently, MacDougall and colleagues [22] have shown the Mindfulness Ambassador Program (MAP) to be acceptable and feasible in FEP, and they are now working on a multicentre, randomised controlled trial (Clinicaltrials.gov identifier NCT03143907) that compare MAP with a waiting list group. Another record by David L. Penn and colleagues compares the Integrated Coping and Awareness Training (I-CAT) with treatment as usual (identifier NCT03067311). Finally, one additional study by Yip Lai King compares Mindfulness-based Cognitive Therapy (MBCT) versus a psychoeducation program in Hong Kong (NCT03501862).

Our team has designed an intervention named SocialMIND, a mindfulness-based social cognition training specifically designed for young people who have suffered an FEP recently. It addresses domains of social cognition that are affected in the FEP [23], such as mental state attributions, emotion recognition, and attributional biases, and integrates them within an acceptance- and mindfulness-based framework. Because these domains account for a significant portion of the variance in social functioning [24], an improvement of real-life outcomes might be expected. We present a protocol for a randomised, controlled trial that is sensitive to three important aspects of the literature on psychosis: first, our sample will be people who have suffered their FEP within the past 5 years; second, a psychoeducational multicomponent intervention (PMI) will be included as an active comparator; and lastly, the primary outcome measure will be personal and social performance. Both SocialMIND and PMI will encompass 8 weekly sessions, 4 bi-weekly sessions, and 5 monthly sessions, and the assessments will be performed after sessions 8, 12, 17, and 3 months later.

## Methods

The aim of this study is to compare the changes in social functioning in a cohort of people who have suffered a first episode of psychosis (FEP) within the past 5 years. They will be assigned to either a mindfulness-based social cognition training (SocialMIND) or a psychoeducational multicomponent intervention (PMI). For that purpose, a parallel-group, single-blind (evaluator), randomised (1:1 ratio), controlled (versus active comparator), superiority, clinical trial will be implemented. The investigation will be conducted at La Paz University Hospital (Madrid, Spain) in accordance with SPIRIT 2013 [25, 26] and CONSORT 2010 [27] statements. The Research Ethics Committee of La Paz University Hospital approved the trial (identifier 4911) and the protocol is available in www.clinicaltrials.gov (identifier NCT03309475). The study is part of the project Environment and Genes in Schizophrenia (AGES-CM 2-CM) (http://www.agescm.es).

### Participants

Eligible participants will be identified by any professional of the Department of Psychiatry, Clinical Psychology and Mental Health of La Paz University Hospital, as well as professionals from other centres of AGES-CM 2-CM. If the participant is already under treatment by a psychiatrist or a psychologist, the treating professional will be contacted to confirm eligibility criteria. The participants will be asked to maintain their treatment as usual with any mental health professional. In order to keep recruitment active, members of the study team will visit every unit of the Mental Health Department every month.

#### Inclusion criteria

1. Age between 18 and 45 years;
2. First hospitalization, first visit to mental health services with positive symptoms, onset of antipsychotic treatment, or first appearance of positive symptoms confirmed by an informant within the period of 5 years prior to enrolment in the study;
3. Signed Informed Consent form

#### Exclusion criteria

1. Other DSM-5 diagnosis, except for nicotine-related disorders and main diagnosis;
2. Clinical Global Impression higher than 5 (“markedly ill”);
3. Intellectual disability plus impaired global functioning prior to disorder onset;
4. Generalised development disorder;
5. Pregnancy
6. Attendance at either mindfulness programs or structured psychoeducational interventions at the time of enrolment

#### Withdrawal criteria

1. Participant’s decision;
2. Hospitalisation in a psychiatric unit;
3. Worsening clinical condition identified by the researchers or the participant’s psychiatrist or psychologist;
4. Attendance at less than 25% of the sessions (less than three weekly-sessions, less than two fortnightly sessions, and less than two monthly sessions)^1^;
5. Attendance at either mindfulness programs or structured psychoeducational interventions during the trial

### Interventions

#### SocialMIND training

SocialMIND is an intervention for people with psychosis designed by the authors. It is a mindfulness-based social cognition training that highlights the importance of cultivating an acceptance-based, non-judgmental approach both towards one’s own experience and to the experience in the interpersonal relationship. It incorporates both formal meditation practices tailored for people with psychosis and sensitive to mental health vulnerabilities and suffering, as well as some social cognition exercises inspired by the Social Cognition and Interaction Training (SCIT) [28]. Practices of formal meditation are based on practices of three formal mindfulness programs: Mindfulness-Based Stress Reduction program (MBSR) [29], Mindfulness-Based Cognitive Therapy (MBCT) [30], and Mindful Self-Compassion program (MSC) [31]. Some of these practices consists of focusing attention on a chosen object either inside (i.e., breathing, body point contact, and body sensations) or outside (i.e., sounds) body scanner, walking meditation, or compassion practices such as loving kindness or soothing touch meditations. SocialMIND teachers are certified teachers of these programs, and recommendations on the adaptation of mindfulness programs for people with psychosis were cautiously looked into [21, 32]. Social cognition training includes attributional bias exercises, mentalising abilities (i.e., theory of mind), or emotion perception and social cues tasks. The full intervention consists of eight weekly sessions, followed by four bi-weekly sessions, and five additional monthly sessions. Each session lasts 90 min and groups are composed of a maximum of 15 participants. The contents of SocialMIND sessions are shown in Table 1.

**Table 1:** SocialMIND training overview

Session	Didactic teaching	Practices	Exercises	Homework
#1. *Awareness of the present-moment*	1. Welcome and presenting to the group: setting and guidelines 2. Introduction to mindfulness	1. *3-min meditation on internal stimuli* (body, feelings, emotions and thoughts) 2. *Mindful movements* (trainees perform a series of movements performed with full awareness) 3. *Shared inquiry: group discussion*	*Raisin exercise: eat a raisin with full awareness*	*Pay attention on daily activities (eating, showering, household chores…)*
#2. *Diversity in perception*	1. Differences between interpreting and describing the experience. 2. Stop before making assumptions on other’s motivations or urges.	1. *3-min meditation on internal stimuli* 2. *Mindful movements* 3. *3-min meditation on breathing, feet, hands or sounds (anchor options)* 4. *Shared inquiry*	1. *The storyteller* (instructors encourage participants to become aware of the human tendency to elaborate narratives from events): describing and interpreting a drawing. 2. *STOP* (instructors and trainees work with this acronym for Stop, Take a breath, Observe and Proceed)	1. *Pay attention on daily activities* 2. *STOP* 3. *Attention on anchor (breathing feet, hands or sounds), when stress is present*
#3. Coping with distress	1. How human beings perceive, elaborate and respond to their experiences (pleasant, unpleasant and neutral) 2. The tendency to avoid unpleasant experiences.	1. *5-min meditation on* body sensations, thoughts and sounds 2. *Walking meditation* 3. *Shared inquiry*	1. *Successive guided reflection* on pleasant and unpleasant experiences 2. *STOP* 3. *Dynamics:* a. *Thoughts: The meditator* (one participant acts as someone who is trying to meditate whilst the others move around him or her and act as distracting thoughts) b. *Primary* versus *secondary distress*	1. *Pay attention on daily activities* 2. *STOP* 3. *Attention on anchor when stress is present* 4. *Walking meditation*
#4. Radical acceptance	1. Acceptance of both pleasant and unpleasant experiences is different from resignation or giving up. 2. Each mental event shall be understood as a product of the mind	1. *5-min meditation on internal stimuli* 2. *Body scan (10 min)* 3. *Mindful movements* 4. *Yes/No meditation* 5. *Shared inquiry*	Emotion recognition in photographs of people’s faces: differences between describing a face and elaborating a story.	1. *Pay attention on daily activities* 2. *STOP* 3. *Attention on anchor when stress is present* 4. *Walking meditation* 5. *Guided practice (voice recorded): 5-min meditation on internal stimuli*; *3 and 5-min meditation* on breathing; *body scan*
#5. Unconditional friendship and compassion	Self-compassion and loving kindness promote health and wellbeing. The importance of being aware of self-criticism and judging attitudes toward oneself and others.	1. *3-min meditation* on internal stimuli 2. *Soothing touch meditation* 3. *Mindful movements* (yoga choices) 4. Loving kindness meditation 5. *Shared inquiry*	1. *The storyteller* 2. Emotion recognition in photographs of people’s faces: differences between describing a face and elaborating a story.	1. *Pay attention on daily activities* 2. *STOP* 3. *Attention on anchor when stress is present* 4. *Walking meditation* 5. *Guided practice (voice recorded): 5-min meditation on internal stimuli*; *3 and 5-min meditation* on *breathing; body scan; soothing touch meditation; loving kindness meditation*
#6. Cultivate the wholesome	Looking for pleasant experiences in order to balance the bias towards the negative experiences.	1. *Body scan: special attention to pleasant sensations.* 2. *Mindful movements* 3. *Loving kindness* meditation 4. *Shared inquiry*	1. Looking for pleasant, nice, beautiful objects, views, sounds… in the room and savoring the experience (body sensations, emotions, feelings, thoughts…) 3. *The storyteller* 4. Emotion recognition in photographs of people’s eyes: differences between describing a face/ eyes and elaborating a story.	1. *Pay attention on daily activities* 2. *STOP* 3. *Attention on anchor (breathing feet, hands or sounds), when stress is present* 4. *Walking meditation* 5. *Guided practice (voice recorded): 5-min meditation on internal stimuli*; *3 and 5-min meditation* on *breathing; body scan; soothing touch meditation; loving kindness meditation* 6. *Looking for pleasant experiences*
#7. Relationship and connection	1. The connection. Human beings are social beings and we need to connect in a safe environment with another person. 2. Pause when tension arises. 3. Introduce the mindful dialogue	1. *Body scan* 2. *Mindful movements* 3. *Loving kindness* meditation 4. *Shared inquiry*	1. Interpersonal exercise of loving kindness and compassion 2. Mindful dialogue 3. Emotion recognition in photographs of people’s eyes: differences between describing a face/ eyes and elaborating a story.	1. *Pay attention on daily activities* 2. *STOP* 3. *Attention on anchor when stress is present* 4. *Walking meditation* 5. *Guided practice (voice recorded): 5-min meditation on internal stimuli*; *3 and 5-min meditation* on *breathing; body scan; soothing touch meditation; loving kindness meditation* 6. *Looking for pleasant experiences*
#8. To live in balance	1. Equanimity: being able to be with the pleasant and the unpleasant experiences, without pushing anything out of consciousness 2. Delivery of certificates: 8-weeks SocialMind training certificate	*50 min of continuum practice:* 1. *Body scan* 2. *Walking meditation* 3. *Meditation on anchor options* 4. *Mindful movements* 5. *Soothing touch* meditation 6. *Loving kindness* meditation *Shared inquiry: group discussion*	1. *The storyteller* 2. Emotion recognition in photographs of people’s eyes: differences between describing a face/ eyes and elaborating a story. 3. Letter to yourself: what you have learned and what are your proposals at the end of the program	1. *Pay attention on daily activities* 2. *STOP* 3. *Attention on anchor (breathing feet, hands or sounds), when stress is present* 4. *Walking meditation* 5. *Guided practice (voice recorded): 5-min meditation on internal stimuli*; *3 and 5-min meditation* on *breathing; body scan; soothing touch meditation; loving kindness meditation* 6. *Looking for pleasant experiences*
#9-12. Consolidation sessions and #12-15. Integration (in daily life) sessions (Contents of consolidation sessions in an interactive way and focalizing in participants´ choices)	It emerges from the experience of the participants and is presented in an interactive way: 1. Emphasis on interpersonal practice 2. STOP 3. Looking for pleasant experiences 4. Equanimity 5. Friendship and compassion towards connection with myself and other beings 6. Be aware of jumping to conclusions (stories)	Possibilities: 1. *Meditation on internal stimuli* (body, feelings, emotions and thoughts) 2. *Breathing meditation* 3. *Walking meditation* 4. *Meditation on breathing, feet, hands or sounds (anchor options)* 5. *Body scan* 6. *Mindful movements* 7. *Soothing touch* meditation 8. *Loving kindness* meditation *Shared inquiry*	1. Interpersonal sharing of experiences 2. Emotion recognition in people’s faces (group’s couples): differences between describing a face and elaborating a story.	1. *Pay attention on daily activities* 2. *STOP* 3. *Attention on anchor (breathing feet, hands or sounds), when stress is present* 4. *Walking meditation* 5. *Guided practice (voice recorded): 5-min meditation on internal stimuli*; *3 and 5-min meditation* on *breathing; body scan; soothing touch meditation; loving kindness meditation* *Looking for pleasant experiences*

#### Psychoeducational multicomponent intervention (PMI)

PMI is a group intervention for people with a FEP that has been developed and standardised by the authors. It incorporates several components of multimodal early intervention programs with empirical support, such as psychoeducation for patients and for their families, medication review, vocational and educational counselling, crisis management and relapse prevention [13]. Two psychiatrists will lead the groups, but the program is well-structured and other mental health professionals such as psychologists, mental health nurses, or mental health residents can lead the PMI after some training. The full intervention consists of eight weekly sessions, followed by four bi-weekly sessions, and five additional monthly sessions. Each one lasts 90 min and groups are composed of a maximum of 15 participants. The weekly sessions (1st to 8th) focus on providing information about psychosis: signs and symptoms, possible underlying diagnosis, pharmacological treatments and their side effects, and psychosocial therapies. During bi-weekly sessions 9th to 12th and during monthly sessions 13th to 15th, participants and therapists design an individualised well-being plan focusing on self-care abilities, coping strategies and practice of social skills. In these sessions, an individual crisis management protocol is developed for each participant. Lastly, participants are asked to invite their family, friends, or any significant person to the two final sessions (16th and 17th), in order to give them information about psychosis and to empower them as key agents for a successful implementation of the well-being plan. Table 2 shows an example of one of the sessions.

**Table 2:** Psychoeducational multicomponent intervention: Contents of session 2 (“Understanding psychosis”)

Structure	Contents	Dynamics	Duration
Welcome and small talk	Small talk about the week	Conversation	10 min
Summary of the previous session	Group setting and norms; overview of Mental Health Services in the Region of Madrid; questions and comments	Oral exposition	20 min
Presentation of the topic of the session	1. What is psychosis. Signs and symptoms 2. The diathesis-stress model: risk and protective factors 3. The phases of the psychotic process	Oral exposition. Q&A	40 min
Questions, comments and debate	Participants are invited to express their doubts about the topic and to share any experience related to it	Conversation. Q&A	20 min
Closure	Brief summary of the session Brief introduction to the next session Homework	Oral exposition. Distribution of materials	10 min

The interventions SocialMIND and PMI will be scheduled in the evenings to reach participants who are studying or working in the morning; nonetheless, minor modifications will be made if consensus is achieved (e.g., start 10 min later so one participant can arrive on time). Two co-therapists will conduct the sessions and one research assistant will be in charge of sending reminders for each session (text messages or phone calls depending on the participant’s preference). Although both interventions are standardised, some contents and practices of both programs might be modified based on clinical decisions (e.g., modify the duration of a formal meditation practice, adapt the content of a psychoeducational session to the period of time since the first episode of psychosis, etc.). In order to keep these minor protocol variations to a minimum, sessions will be video recorded to check if the professionals comply with the intervention manual.

### Outcomes and measurements

Most outcomes will be measured at five time periods: before randomisation (t_0_, baseline), after weekly sessions (t_1_, 8 weeks), after bi-weekly sessions (t_2_, 16 weeks), after monthly sessions (t_3_, 36 weeks) and 12 weeks after end of the intervention (t_4_, 48 weeks). Weekly and bi-weekly sessions are the most intensive part of the treatment, whilst monthly sessions are booster sessions; thus, change between t_0_ and t_2_ is set as the main outcome across the different domains. Table 3 shows the participant’s timeline.

**Table 3:** Participant’s timeline

	Study period					
	Enrolment	Allocation	Post-allocation			Close-out
Timepoint	t_−1_	0	t_1_	t_2_	t_3_	t_4_
Enrolment						
Eligibility screen	X					
Informed consent	X					
Allocation		X				
Interventions						
SocialMIND			W	B	M	
PMI			W	B	M	
Assessments						
Baseline	X					
Demographic						
Duration of untreated illness						
Duration of untreated psychosis						
Number of hospitalizations						
Diagnosis						
Psychosocial and pharmacological treatment						
Functional outcomes	X		X	X	X	X
Social functioning						
General functioning						
Clinical outcomes	X		X	X	X	X
Positive syndrome						
Negative syndrome						
General psychopathology						
Depressive symptoms						
Anxiety symptoms						
Adverse events						
Biological outcomes	X			X	X	
Cytokines						
Antioxidant status						
Oxidative/nitrosative stress						
Social cognition outcomes	X			X	X	
Theory of mind						
Attributional style						
Emotion recognition						
Cognitive outcomes	X			X	X	
Processing speed						
Working memory						
Vigilance						
Emotional intelligence						
Other outcomes	X		X	X	X	
Mindful attention and awareness						
Adherence to SocialMIND manual^a^						

*PMI* psychoeducational multicomponent intervention, *W* weekly sessions, *B* bi-weekly sessions, *M* monthly sessions

^a^ Only from t_1_ to t_3_

### Baseline measures

#### Sociodemographic measures

Age, gender, marital status, educational level, job status and parental level of education and parental job status will be codified.

#### Clinical measures

History of hospitalisations since the FEP, duration of untreated illness, duration of untreated psychosis, significant life events, and DSM-5 diagnosis will be registered. Other pharmacological and non-pharmacological interventions as well as current medication and psychosocial care will be checked.

### Primary outcome variable

#### Social functioning

Change in social functioning between t_0_ and t_2_ will be measured using the Personal and Social Performance (PSP) [33] scale for schizophrenia. This scale explores four domains of social functioning, namely self-care, social relationships, social activities, and disruptive and aggressive behaviour. After the semi-structured interview, a final score (ranging from 1 to 100) is obtained, with higher values indicating better performance. The Spanish version of the PSP [34] is reliable and presents high internal consistency (Cronbach’s alpha = 0.87), and excellent test-retest reliability (*r* = 0.98), and good construct validity (one single component explains 73% of the variance in social functioning). The mean of the PSP-T is 50.3 and the standard deviation is 18.6 points, with an increment of 15 points being considered clinically significant.

### Secondary outcome variables

#### Social functioning

Change in social functioning between t_0_ and t_2_, and between the remaining endpoints (t_1_, t_3_ and t_4_) will be assessed with the PSP. Changes from t_0_ to t_2_ in its final score are set as the primary outcome measure; changes between the remaining endpoints (t_1_, t_3_ and t_4_), and changes in subscales (self-care, social relationships, social activities, and disruptive and aggressive behaviour) are secondary outcome measures. Subscales provide ordinal data that range from “absent” to “very severe”, with higher values indicating worse performance.

#### General functioning

Change in general functioning between t_0_ and t_2_, and between the remaining endpoints (t_1_, t_3_ and t_4_) will be assessed with the General Assessment of Functioning (GAF) [35, 36] scale. It provides a score from 1 to 100, with higher values indicating better general functioning. It has good inter-rater reliability and is also associated with symptoms and social functioning in people with schizophrenia [37]. The evaluator completes this scale after a semi-structured interview exploring clinical and functional outcomes.

#### Positive and negative syndrome

Change in positive and negative symptoms, and in general psychopathology, between t_0_ and t_2_, and between the remaining endpoints (t_1_, t_3_ and t_4_), will be assessed with Positive and Negative Syndrome Scale (PANSS) [38] for schizophrenia. Values range from 1 (“absent”) to 7 (“extreme”), and final scores range from 7 to 49 for positive (PANSS-P) and negative (PANSS-N) syndromes, and from 16 to 112 for general psychopathology. A semi-structured interview is conducted to rate each dimension. Subscales of Spanish version are strongly associated with the original version (*r* = 0.92 for PANSS-P and *r* = 0.83 for PANSS-N), with item correlations ranging from *r* = 0.64 to *r* = 0.97 and high inter-rater reliability (*r* = 0.81) [39].

#### Depressive symptoms

Change in depressive symptoms between t_0_ and t_2_, and between the remaining endpoints (t_1_, t_3_ and t_4_), will be assessed with Calgary Depression Scale for Schizophrenia (CDSS) [40]. A semi-structured interview is conducted and a final score between 0 and 36 is obtained, with lower values indicating fewer symptoms. Spanish version of CDSS has high internal consistency (Cronbach’s alpha = 0.83) and high inter-rater reliability (ICC > 0.70); it also discriminates between depressed and non-depressed people with SSD [41].

#### Anxiety symptoms

Change in anxiety symptoms between t_0_ and t_2_, and between the remaining endpoints (t_1_, t_3_ and t_4_), will be assessed with self-reported Beck Anxiety Inventory (BAI) [42]. It is a 21-item scale and participants rate how affected have they be by a list of anxiety symptoms during the last week in a 0 to 3 Likert scale. Scores range between 0 (minimum, no anxiety) and 84 (maximum, extreme anxiety). Spanish version [43] has high internal consistency (Cronbach’s alpha = 0.90) and discriminates between people with and without anxiety disorders [43, 44].

#### Screening of bipolar disorder

Change in manic and depressive symptoms between t_0_ and t_2_, and between the remaining endpoints (t_1_, t_3_ and t_4_), will be rate by the assessor with Clinical Global Impression for Bipolar Disorder (CGI-BD) [45]. Two Likert scales rate depressive and manic symptoms in the last week, ranging from 1 (“normal”) to 7 (“extreme”); one additional 7-points Likert scale evaluates general severity in the last year. It is better than the original CGI for the assessment of bipolar disorder and has good inter-rater reliability [45]. Scores are based on the assessor’s clinical impression after a semi-structured interview.

#### Adverse events

Visits to the emergency room, hospitalizations, and treatment discontinuation will be registered in detail from baseline to the end of the study.

### Tertiary outcome variables

#### Social cognition (mental state attribution)

Change in theory of mind between t_0_ and t_2_, and between the remaining endpoints (t_1_, t_3_ and t_4_), will be assessed with the 5-item version of the Hinting Task [46, 47] and with the revised version of Eyes Test [48, 49]. The Hinting Task consists of five stories where a character insinuates that he or she wants the other to do something. The assessor reads the situations out loud to the participant, whose response is written down literally. Scores range from 2 to 0 points for each item, and final score ranges from 0 (worst performance), to 10 (best performance). Internal consistency (Cohen’s alpha = 0.78), test-retest stability (Cohen’s kappa = 0.83), and inter-rater reliability (Cohen’s kappa = 0.94) are good in the Spanish version, which accurately discriminates between people with schizophrenia and healthy controls [50]. The revised version of the Eyes Test comprises 36 pictures of the eyes area expressing different emotions, so it recruits emotion recognition abilities as well [51]. There are four response options for each item and only one of them is correct. One point is given for each correct response, so final score ranges from 0 to 36. It has been adapted to Spanish population with a good test-retest reliability (ICC = 0.76) [52].

#### Social cognition (emotion recognition)

Change in emotion recognition between t_0_ and t_2_ and between the remaining endpoints (t_1_, t_3_ and t_4_), will be assessed with the Penn Emotion Recognition Test (ER-40) [53] consisting of 40 pictures of actors and actresses performing facial expressions for different basic emotions. Participants are always given the same five response options: happy, sad, anger, fear, and no emotion. A Spanish version is not available, and translation of the emotions’ names matched the translation of a similar task [54].

#### Social cognition (attributional style)

Change in attributional style between t_0_ and t_2_, and between the remaining endpoints (t_1_, t_3_ and t_4_) will be assessed with the Ambiguous Intentions Hostility Questionnaire (AIHQ) [55]. Participants read fifteen stories that are either accidental, intentional, or uncertain (i.e., “you walk past a bunch of teenagers at a mall and you hear them start to laugh”). After reading each one of the stories, they are asked the following questions: a) why did the character do what he or she did?, b) did he or she do that on purpose?, c) how angry does it make you feel?, d) would you blame the character?, e) what would you do if you were in that situation? In items a) (hostility bias) and e) (aggression bias), participants are asked to write down their responses, which will be scored by two independent evaluators according to AIHQ scoring criteria; for item b) (intention index), a six-point Likert scale is provided; for items c) (blame index) and d) (anger index), a five-point Likert scale is provided. Five different indexes and one extra compound score are obtained. It is not adapted to the Spanish population.

#### Mindful attention and awareness

Change in mindful attention and awareness between t_0_ and t_2_, and between the remaining endpoints (t_1_, t_3_ and t_4_), will be assessed with the Mindful Attention and Awareness Scale (MAAS) [56]. It is a self-reported measure of mindful disposition and its scores range from 0 to 90, with higher values indicating that the individual is more disposed to be aware and pay attention. The Spanish version has good internal consistency (Cohen’s alpha = 0.89) and high temporal stability (*r* = 0.823), with low sensitivity to change after mindfulness trainings (*r* = 0.79) [57].

#### Neurocognition

Change in neurocognition between t_0_ and t_2_, and between the remaining endpoints (t_1_, t_3_ and t_4_), will be assessed with five tasks of the Matrics Consensus Cognitive Battery (MCCB) [58]. Specifically, we included one processing speed index (Brief Assessment of Cognition in Schizophrenia: Symbol Coding [BACS]); four vigilance indexes of Continuous Performance Test - Identical Pairs (CPT-IP); and one verbal (Letter and Number Span [LNS]) and one visuospatial (Wechsler Memory Scale: Spatial Span [WMS-SS]) working memory tasks. Additionally, MCCB includes the Managing Emotions task of the Mayer-Salovey-Caruso Emotional Intelligence Test (MSCEIT). Standardized scores will be obtained through international MCCB scoring software, corrected for age, gender, and educational level.

#### Biological outcomes

Change in cytokines (IL1β, IL6 y TNFα), antioxidant status (TAS, CAT, SOD, GPx), and indicators of oxidative/nitrosative stress (TBARS) will be assessed between t_0_ and t_2_, and between t_0_ and t_3_. Specific assay kits will be used.

#### SocialMIND teachers’ checklist

This instrument consists of two parts. The first is inspired by the assessment of protocol compliance in Social Cognition and Interaction Training (SCIT) [28]. Raters must check if the teachers adhere to the SocialMIND manual and complete 8 items that range from 0 to 2 points, with higher values indicating more adherence. The second part is the Mindfulness-based Interventions: Teaching Assessment Criteria (MBI:TAC) [59], which comprises six domains that should be addressed in each session, such as embodiment of mindfulness, correct guidance, or holding the group environment. Teachers can obtain a score between 1 (“Incompetent: Absence of key features or highly inappropriate performance”) and 6 points (“Advanced: Excellent teaching practice, or very good even in the face of participant difficulties”). Assessments were made after checking video recordings of the sessions.

#### Psychoeducational multicomponent intervention checklist

This instrument is also inspired in the assessment protocol of compliance to the SCIT, and the evaluators must complete 8 items that range from 0 to 2 points, with higher values indicating more adherence [28].

### Assessment procedure

The assessment will be separated into two or three sessions according to the participant’s preference. If the participant wants to divide the assessment in two sessions, blood tests and clinical measures, will be performed in the first appointment, and neurocognition and social cognition tasks in the second appointment. If the participant prefers to divide the assessment in three sessions, the first one will consist of blood tests and clinical measures, the second one will be the social cognition battery, and the last one will be the neurocognitive assessment. Morning and evening appointments will be available so participants can choose which one fits them better. For neurocognition tasks, if the baseline assessment is made in the morning, upcoming appointments will also be scheduled in the morning (in case of a baseline assessment in the evening, subsequent appointments will be made in the evening).

### Assignment of interventions

Participants will receive a unique AGES-Mind identifier (e.g., AM-99) that will ensure his or her anonymity during the trial. The day before the interventions start, a research assistant will perform randomisation using TeamMaker™, a free software available from http://chir.ag/projects/team-maker/. Participants’ identifiers will be introduced, and two teams will be formed (SocialMIND and PMI). A .csv file with treatment allocation will be obtained and sent to the therapists, and the research assistant will call the participants and inform them about treatment allocation.

Such different behavioural interventions do not permit to mask the allocation for neither the research assistant, nor the therapists, or the participants; but the outcomes evaluator and data analyst will be blind to treatment allocation. It is difficult to ensure masking for a clinical trial that lasts a whole year, so the evaluators will not be responsible for contacting participants to perform the assessments; instead, they will be waiting for the participants in a room, and the participants will be advised twice about the importance of not revealing intervention assignment. For biological variables, the nurse performing the extraction will be blind to treatment assessment, but he will be assisted by a researcher who will not be. Materials for blood samples will not contain any information that reveals treatment allocation. Data analysts will receive a database with a dichotomous variable called “intervention arm” with two values (“1” and “2”) that will be randomly chosen too.

If unblinding happened to one of the evaluators, he or she will inform one of the leading investigators and the assessment will be repeated by another evaluator. The non-blind evaluator will not evaluate this participant again.

### Data collection, sample size calculation and statistical analysis

Different evaluators will collect the data across the time points with the supervision of two research assistants and the two lead investigators. Evaluators will attend at least 1 day of training to become familiarised with the instruments, especially the rating scales and the neurocognitive battery. For self-reported measures (e.g., questionnaires, inventories) there will be no specific training. The research assistant in charge of data entry will check for any queries and solve them with the evaluator; then the assistant will enter data into the database, and ranges will be checked for each variable to detect possible errors. Database will be stored in a private server and in a hard disk in La Paz University Hospital. Participants who stop coming to the sessions will be asked to attend an assessment session and primary and secondary outcome data will be collected. They will be contacted again when it is time for the assessment in order to reduce missing data; in case they are not available or decide not to come, the last observation will be carried forward to the remaining time points.

For the primary outcome measure, an analysis of covariance (ANCOVA) will be performed to explore differences between the two interventions in the final score of the Personal and Social Performance Scale (PSP-T) after bi-weekly sessions (t_2_). PSP-T baseline score (t_0_) will be introduced as a covariate in the ANCOVA model. A 15-point increment is considered clinically significant [24] and corresponds to a moderate effect size (Cohen’s f = .40). In order to detect a change from t_0_ to t_2_ with a 90% of probability (1-ß = 0.90) and a type I error of 5% (α = 0.05), 68 participants should enrol in the trial. Considering an attrition rate around 15%, a final sample of 80 participants would be needed.

Goodness of fit indexes will be obtained to explore the distribution of all variables. Baseline demographic characteristics, biological outcomes, clinical variables, and cognitive scores will be assessed to test if groups are equivalent: t-tests will be used for interval and ratio variables, Mann-Whitneys U test for ordinal variables, and Pearson’s chi-squared tests for nominal variables. Missing values will be imputed with the last observation carried forward (LOCF) method. Intervention effects over interval and ratio variables will be assessed with a 2 (Intervention arms: PMI, SocialMIND) × 5 (Time: t_0_, t_1_, t_2_, t_3_, t_4_) ANCOVA, with baseline observation (t_0_) introduced as a covariate for t_1_ and t_2_, and the previous time-point for t_3_ (t_2_ as a covariate) and for t_4_ (t_3_ as a covariate). Outcomes will be explored to check for violations of any ANCOVA assumptions. For dichotomous or dichotomised variables, number needed to treat (NNT) and risk ratios will be provided. Data will be analysed following a modified intention-to-treat model which will exclude participants who attend less than 3 weekly-sessions because they cannot be considered to have received the intervention. Standard intention-to-treat and per-protocol analyses will also be reported. Two-tailed tests will be carried out with alfa set at 0.05. Data will be introduced in the database by a research assistant with knowledge of treatment allocation and analysed by a statistician blind to treatment allocation.

### Monitoring

Risk of harm needs to be monitored during a clinical trial. The Research Ethics Committee will receive a report either once a year -if no adverse effects are detected-, or immediately -if any adverse event is detected. Moreover, only participants who are referred by their psychiatrists or psychologists are enrolled, so feedback mechanisms will be implemented between the professionals and the study team (e.g., e-mails, phone calls, visits, etc.). Evaluators will also ask for any adverse event detected since the previous visit.

One important risk of bias might happen when the study team decides to conclude the trial. Solutions such an external endpoint adjudication committee have been proposed. For our study, we have performed an a-priori sample size calculation, which has been made public in www.ClinicalTrials.gov and in this paper. Interim analyses will be performed, but they will not condition the decision to terminate the trial. Moreover, this protocol is being funded by the European Regional Development Fund (ERDF) and monitored by a panel of experts of the Institute of Health Carlos III (ISCIII) (identifier PI 17/00768).

One last point of great importance for behavioural interventions is the compliance with the intervention protocol. As described above, adherence to SocialMIND and PMI intervention manual will be monitored, and sessions will be recorded.

### Ethics and dissemination

The study was approved by the Research Ethics Committee of La Paz University Hospital, with the identifier 4911. Three amendments were made before final approval. Every participant will sign two informed consents; the first one includes general aspects of the investigation and session video recordings, and the second one is specific for biological samples. These biological samples will be registered, processed and stored in the Hospital La Paz-IdiPAZ Biobank. All the information provided by the participants will be codified twice: the first code will identify every outcome variable (including biological outcomes), and the second one will be provided by the Biobank and will be exclusive for biological variables. Datasheets will not include the name, the surname or the address of any of the participants. The correspondence between participants’ identifier and their personal data will be codified in a data spreadsheet file which will be stored by duplicate: one copy in a Network Attached Storage (NAS) of the Madrilenian Department of Health and the other in an external hard disk in a private office of La Paz University Hospital. Backups of video recordings will be made after each session and will be stored in this hard disk; then, the file will be erased from the camera. Participants will also be offered to participate in the group to which they have not been assigned after study completion (t_4_). Results will be made public in www.clinicaltrials.gov and in scientific communications (conferences, articles, posters, presentations…), and priority will be given to open-access journals. A final plain-language report will be given to each participant who is interested, and to the professionals who refer participants. The dataset supporting the conclusions of the randomised controlled trial will be available from the corresponding author on request.

## Discussion

We present the study protocol for a randomised controlled trial that explores the effectiveness of SocialMIND, the first mindfulness-based social cognition group training developed for people with psychosis. Both SocialMIND and the psychoeducational multicomponent intervention (PMI) that serves as an active comparator have an intensive phase of 16 weeks -with 8 weekly sessions followed by 4 bi-weekly sessions-, and an extensive phase of 20 weeks with 5 monthly sessions.

Manualised Mindfulness-based interventions (MBI) such as the Mindfulness-based Stress Reduction program (MBSR) or the Mindfulness-based Cognitive Therapy (MBCT) lasts 8 weeks; however, people with higher levels of suffering and severe symptoms might benefit from longer programs. Hence, although primary and secondary outcomes will be measured after weekly sessions (t_1_), our hypothesis is that the training will be effective after completing the whole intensive phase (t_2_), so statistical analysis for our primary hypothesis will consider the change from baseline (t_0_) to t_2_. An additional period of five monthly sessions is proposed in order to tests if changes are maintained, so exploring the difference between the time point after monthly sessions (t_3_) and t_2_ will be of great interest.

The main obstacle of the study will be the recruitment of the 80 participants. This a priori sample size calculation is based on the Spanish validation of the Personal and Social Performance (PSP) scale, but the variability of our scores -and hence the required sample size- might be lower as our group will be more homogeneous in terms of duration of the illness, age, diagnosis, etc. There are many problems inherent in the use of patient-reported outcome measures (PROM) in clinical trials [60], but social functioning is a domain hardly measurable with instruments that are not self-reported; considering this, our study will adhere to the SPIRIT guidelines extension to PROM [26]. Moreover, the DELTA (Difference Elicitation in TriAls) project proposes that the primary outcome should be of relevance to at least one key stakeholder group [61], and improvements in social functioning are indeed crucial for patients, health professionals, and the Administration, as it is directly associated with real-life outcomes. In the case that social functioning would indeed increase after the intervention, a moderator analysis which incorporates different measures of social cognition and neurocognition would be of great interest. As we are conservative with our sample size estimation, these factors will only be explored as tertiary outcomes, leaving further analysis for upcoming investigations.

Our goal is ambitious. First, we expect to improve real-life outcomes, which is far more difficult than proving a reduction of symptoms or an increase of social cognition scores; second, we expect this improvement to be equal to or higher than the improvement in the active comparator arm; and lastly, we will include a cohort of people who rarely adhere to therapeutic programs [5]. Regardless of these difficulties, a patient-centered intervention such as SocialMIND may help young people who have suffered a FEP to create unique, meaningful narratives of their experience, and to disengage from it and the suffering that it entails. In the words of Rufus May, “if we are able to achieve some detachment from our beliefs in the knowledge they are just one way of seeing the world, we become more aware that ‘the map’ (i.e. our beliefs) is not the territory; and that different maps tell us about different aspects of the territory. It seems helpful to adopt a relational perspective towards our beliefs, thoughts and perceptions so that we both detach from them and try to relate to them” [62].

## Data Availability

The datasheets and the manual for both interventions will be available from the corresponding author.

## Abbreviations

AGES-CM 2-CM: Ambiente y Genes en Esquizofrenia – Comunidad de Madrid [Genes and Environment in Schizophrenia – Region of Madrid]
AIHQ: Ambiguous Intentions Hostility Questionnaire
ANCOVA: Analysis of Covariance
BACS: Brief Assessment of Cognition in Schizophrenia
BAI: Beck Anxiety Inventory
CAT: Catalase
CDSS: Calgary Depression Scale for Schizophrenia
CGI-BD: Clinical Global Impression for Bipolar Disorder
CPT-IP: Continuous Performance Test – Identical Pairs
DELTA: Difference Elicitation in TriAls
ER-40: Emotion Recognition Test
ERDF: European Regional Development Fund
FEP: First episode of psychosis
GAF: General Assessment of Functioning
GPx: Glutathione perioxidase
I-CAT: Integrated Coping and Awareness Training
IdiPAZ: Instituto de Investigación Sanitaria del Hospital Universitario La Paz [Hospital La Paz Institute for Health Research]
IL: Interleukin
ISCIII: Instituto de Salud Carlos III [Institute of Health Carlos III]
LNS: Letter and Number Span
MAAS: Mindful Attention and Awareness Scale
MAP: Mindfulness Ambassador Program
MBCT: Mindfulness-Based Cognitive Therapy
MBI:TAC: Mindfulness-Based Interventions: Teaching Assessment Criteria
MBSR: Mindfulness-Based Stress Reduction program
MCCB: MATRICS Consensus Cognitive Battery
MSC: Mindful Self-Compassion program
MSCEIT: Mayor-Salovey-Caruso Emotional Intelligence Test
NAS: Network Attached Storage
NNT: Number needed to treat
PANSS: Positive and Negative Syndrome Scale for Schizophrenia
PMI: Psychoeducational multicomponent program
PROM: Patient-reported outcome measures
PSP: Personal and Social Performance scale
SCIT: Social Cognition and Interaction Training
SOD: Superoxide dismutase
TAS: Total antioxidant status
TBARS: Thiobarbituric and reactive substances
TNF: Tumor necrosis factor
WMS-SS: Wechsler Memory Scale – Spatial Span

## Footnotes

1 Participants could keep attending the groups if they wanted to and if the therapists agreed
